# Differentiation of Idiopathic Pulmonary Fibrosis from Connective Tissue Disease-Related Interstitial Lung Disease Using Quantitative Imaging

**DOI:** 10.3390/jcm10122663

**Published:** 2021-06-17

**Authors:** Jonathan H. Chung, Ayodeji Adegunsoye, Brenna Cannon, Rekha Vij, Justin M. Oldham, Christopher King, Steven M. Montner, Prahasit Thirkateh, Scott Barnett, Ronald Karwoski, Brian J. Bartholmai, Mary Strek, Steven D. Nathan

**Affiliations:** 1 Department of Radiology, The University of Chicago Medicine, Chicago, IL 60637, USA; smontner@radiology.bsd.uchicago.edu; 2 Department of Medicine, The University of Chicago Medicine, Chicago, IL 60637, USA; aadegunsoye@medicine.bsd.uchicago.edu (A.A.); rekhavij1@gmail.com (R.V.); mstrek@medicine.bsd.uchicago.edu (M.S.); 3 Inova Advanced Lung Disease and Transplant Program, Inova Fairfax Hospital, Falls Church, VA 22042, USA; bc9445@pcom.edu; 4 Department of Medicine, The University of California Davis, Sacramento, CA 95616, USA; joldham@ucdavis.edu; 5 Advanced Lung Disease and Transplant Clinic, Inova Fairfax Hospital, Falls Church, VA 22042, USA; Christopher.King@inova.org; 6 Chicago Medical School at Rosalind Franklin University of Medicine and Science, North Chicago, IL 60064, USA; prahasit.thirkateh@my.rfums.org; 7 Inova Heart and Vascular Institute, Inova Fairfax Hospital, Falls Church, VA 22042, USA; scottdb65@gmail.com (S.B.); Steven.Nathan@inova.org (S.D.N.); 8 Department of Physiology and Biomedical Engineering, Mayo Clinic, Rochester, MN 55902, USA; karwoski.ronald@mayo.edu; 9 Department of Radiology, Mayo Clinic, Rochester, MN 55902, USA; Bartholmai.Brian@mayo.edu

**Keywords:** connective tissue disease, idiopathic pulmonary fibrosis, usual interstitial pneumonia, image interpretation, computer-assisted, multidetector computed tomography

## Abstract

A usual interstitial pneumonia (UIP) imaging pattern can be seen in both idiopathic pulmonary fibrosis (IPF) and connective tissue disease-related interstitial lung disease (CTD-ILD). The purpose of this multicenter study was to assess whether quantitative imaging data differ between IPF and CTD-ILD in the setting of UIP. Patients evaluated at two medical centers with CTD-ILD or IPF and a UIP pattern on CT or pathology served as derivation and validation cohorts. Chest CT data were quantitatively analyzed including total volumes of honeycombing, reticulation, ground-glass opacity, normal lung, and vessel related structures (VRS). VRS was compared with forced vital capacity percent predicted (FVC%) and percent predicted diffusing capacity of the lungs for carbon monoxide (DLCO%). There were 296 subjects in total, with 40 CTD-ILD and 85 IPF subjects in the derivation cohort, and 62 CTD-ILD and 109 IPF subjects in the validation cohort. VRS was greater in IPF across the cohorts on univariate (*p* < 0.001) and multivariable (*p* < 0.001–0.047) analyses. VRS was inversely correlated with DLCO% in both cohorts on univariate (*p* < 0.001) and in the derivation cohort on multivariable analysis (*p* = 0.003) but not FVC%. Total volume of normal lung was associated with DLCO% (*p* < 0.001) and FVC% (*p* < 0.001–0.009) on multivariable analysis in both cohorts. VRS appears to have promise in differentiating CTD-ILD from IPF. The underlying pathophysiological relationship between VRS and ILD is complex and is likely not explained solely by lung fibrosis.

## 1. Introduction

Idiopathic pulmonary fibrosis (IPF) is the most common form of the idiopathic interstitial pneumonias as well as the most common cause of pulmonary fibrosis in most populations. Previously, discerning the exact diagnosis was thought to be largely academic given that management of most types of pulmonary fibrosis was similar. However, with the recent availability of antifibrotic agents for IPF as well as data showing that immuosuppression in IPF is associated with worse outcomes, an accurate diagnosis in the setting of pulmonary fibrosis has become essential [1,2,3].

The imaging and histological correlate in IPF is a pattern that has been termed usual interstitial pneumonitis (UIP). However, a UIP pattern can also be seen with connective tissue disease-related interstitial lung disease (CTD-ILD), particularly with rheumatoid arthritis. This is especially problematic in cases of CTD that present initially as ILD, in the absence of systemic manifestations [4,5] and, therefore, mimic IPF. Although there are certain CT findings that are suggestive of CTD-ILD in the setting of UIP, these specific findings are present in a minority of cases [6,7,8]. In addition, HRCT interpretation is generally hampered by substantial inter-reader variation [9,10,11]. An automated tool that could reliably analyze complex imaging ILD data from chest CT scans and provide decision support to favor a specific diagnosis would be highly valuable, particularly outside of academic centers where there is a lower level of ILD expertise in chest imaging interpretation.

**Figure 1:**
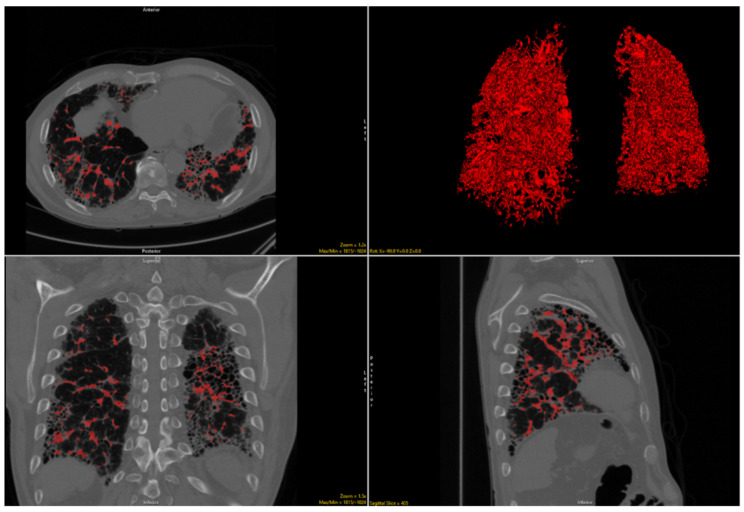
Delineation of volume-related structures on noncontract chest CT images and 3D reconstruction in a patient with idiopathic pulmonary fibrosis.

**Figure 2:**
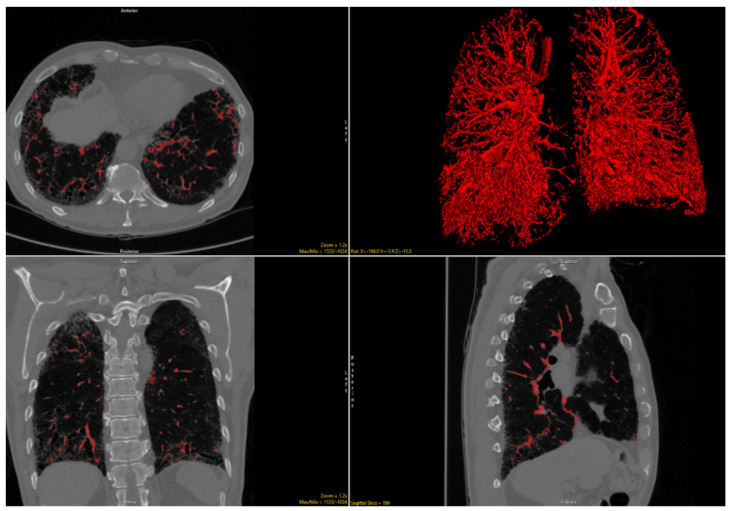
Delineation of volume-related structures on noncontract chest CT images and 3D reconstruction in a patient with connective tissue disease-related interstitial lung disease.

The quantitative imaging tool known as Computer-Aided Lung Informatics for Pathology Evaluation and Rating (CALIPER) has been repeatedly shown to predict patient outcomes and has been associated with pulmonary function [12,13,14,15,16,17,18,19]. A CALIPER quantitative variable strongly associated with patient prognosis across multiple diagnoses is vessel-related structure (VRS) volume (Figure 1 and Figure 2). In the anatomic segmentation process of the CALIPER software, the vessel-related structures are detected and extracted algorithmically for the purpose of exclusion of these branching structures from the other parenchymal feature classification (such as normal lung, ground-glass opacity, or reticular densities) of the volumetric data. Surprisingly, the VRS has been shown to be an independent marker of patient outcomes in ILD [12,17,19]. Since CTD-ILD has superior survival compared to IPF, we postulated that VRS as measured by CALIPER may be able to differentiate CTD-ILD from IPF even in the setting of UIP [6,20,21,22]. The purpose of this multicenter study was to assess whether quantitative imaging data differed across IPF and CTD-ILD in the setting of UIP.

## 2. Materials and Methods

### 2.1. Subjects and Clinical Data

Consecutive adult patients evaluated between 2007 and 2017 at a tertiary ILD medical center with UIP noted either on chest CT scan or surgical lung biopsy with a multidisciplinary diagnosis of CTD-ILD or IPF were included in the study and served as the derivation cohort. Adult patients seen at a second tertiary referral center between 2006 and 2015 with UIP on a chest CT and a multidisciplinary diagnosis of CTD-ILD or IPF were included as an independent validation cohort. CT scans that were reconstructed or acquired with non-HRCT slice thickness (>2 mm) or contained substantial motion or streak artifact were excluded from analysis. The CT scans from the validation cohort were previously used in an earlier study assessing qualitative but not quantitative imaging results [6]. The current study differs from the prior study in that all of the imaging analysis stems from use of the CALIPER quantitative tool, and no imaging data discussed in the previous paper are presented in this study [6]. Age, sex, body mass index, race, smoking history, and functional data were abstracted from the clinical records for both cohorts. This retrospective, two-center HIPAA compliant study was approved by the respective institutional review boards (#17-2684 and #14163A). Written consent was obtained from all patients.

### 2.2. Imaging Analysis

CT scans without axial reconstructed images ≤2 mm were excluded from analysis, leaving 40 CTD-ILD and 85 IPF subjects for formal analysis in the derivation and 62 CTD-ILD and 109 IPF subjects in the validation cohorts, respectively. The axial series with thinnest continuous reconstruction and least sharp algorithm (to minimize artifact from edge-enhancement and noise) were selected for analysis with CALIPER. CALIPER provides automated segmentation and lung analysis including volumetric summation of total pulmonary reticulation, ground glass opacity, honeycombing, normal lung, low attenuation areas and VRS; zonal and axial distributions within the lung parenchyma are also automatically tabulated as described previously [17].

### 2.3. Statistical Analysis

Continuous data were presented as means with standard deviation or median and interquartile range, as appropriate, and categorical data were presented as counts with proportions. Means were compared using a two-tailed Student’s *t*-test, medians were compared using a Mann–Whitney U test, and proportions were compared using a Chi-square test or Fischer exact test as appropriate. Multivariable analysis of CALIPER-based imaging variables associated with ILD (ground-glass opacity, reticulation, honeycombing, normal lung, and VRS) were performed using standard logistic regression. Receiver operator character curves were used to calculate area under the curve (AUC) statistics. To further clarify the underlying mechanism of the VRS variable, VRS was linearly correlated with percent predicted forced vital capacity (FVC%) and percent predicted diffusing capacity of the lungs for carbon monoxide (DLCO%) using a univariate and multivariable approach. All statistical analyses were performed using Wizard Pro software (version 1.9.22, Evan Miller).

## 3. Results

The derivation cohort consisted of 50 patients with CTD-ILD and 100 patients with IPF, but 25 patients were excluded for inadequate CT image quality (slice thickness above 2 mm, incomplete imaging through the thorax, excessive artifacts), leaving 40 CTD-ILD and 85 IPF subjects for formal analysis. There were 62 CTD-ILD and 109 IPF subjects analyzed in the validation cohort. The derivation cohort CTD subjects were comprised mostly of systemic sclerosis (25/50, 50%), myositis (7/25, 14%), rheumatoid arthritis (6/50, 12%), and mixed connective tissue disease (5/10, 10%) subjects. The validation cohort CTD subjects were comprised mostly of rheumatoid arthritis (26/62, 41.9%), systemic sclerosis (12/62, 19.4%), myositis (9/62, 14.5%), and mixed connective tissue disease (9/62, 14.5%), subjects.

The demographics of included subjects are shown in Table 1 relative to clinical diagnosis. Demographic differences across diagnoses were consistent across centers (Table 1). Subjects with IPF were older and had a greater median pack-year smoking history than CTD-ILD subjects. The majority of CTD-ILD subjects were women, while nearly all IPF subjects were men. Although most CTD-ILD and IPF subjects were white, there was a significantly higher proportion of IPF subjects who were white than CTD-ILD subjects. There was no difference across diagnoses with regard to FVC% and DLCO%.

### 3.1. CALIPER Diagnostic Analysis

#### 3.1.1. Univariate Analysis

In the derivation cohort of patients, all of whom had UIP, univariate analysis showed that the volumes of honeycombing (*p* < 0.001), reticulation (*p* = 0.032), and VRS (*p* < 0.001) were all significantly higher in subjects with IPF than in CTD-ILD (Table 2). There was no difference in total volume of ground-glass opacity or the volume of normal lung between the IPF and CTD-ILD cohorts.

In the validation cohort, univariate analysis showed that the volume of reticulation (*p* = 0.003), VRS (*p* < 0.001), and ground-glass opacity (*p* < 0.001) were significantly higher and that the volume of normal lung (*p* = 0.005) was lower in IPF than in CTD-ILD (Table 2). Total honeycombing was not significantly different, although the difference approached significance (*p* = 0.087).

#### 3.1.2. Multivariable Analysis

For the derivation cohort, multivariable analysis demonstrated that the total VRS volume was significantly higher in IPF than in CTD-ILD (*p* < 0.001), while the total ground-glass opacity volume was significantly lower in IPF than in CTD-ILD (*p* = 0.018) (Table 3). Total volume of honeycombing or total volume of reticulation was not significantly different relative to diagnosis.

**Figure 3:**
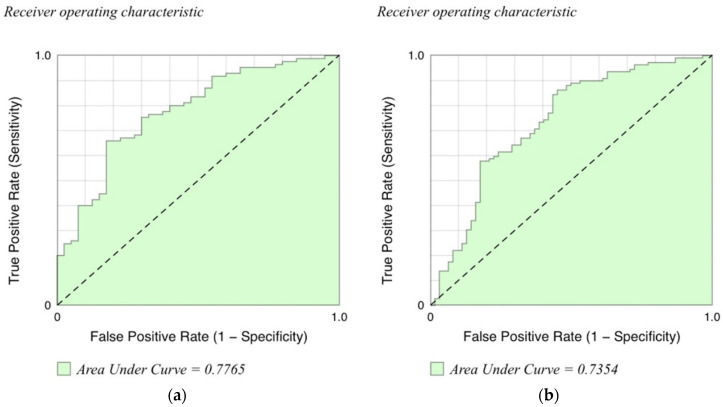
Receiver operating characteristic graphs in the diagnosis of idiopathic pulmonary fibrosis for the derivation (**a**) and validation (**b**) cohorts.

In the validation cohort, total VRS (*p* = 0.047) and total normal lung (*p* = 0.009) volumes were independently associated with an IPF diagnosis (Table 3). There was no difference in total volumes of reticulation, honeycombing, or ground-glass opacity relative to diagnosis. Since VRS was the only variable that was significantly different using multivariate analysis in both cohorts, we generated receiver operating curves using different cutpoints of this variable as a predictor of IPF versus CTD. This resulted in very similar AUCs for the original and validation cohorts of 0.77 and 0.73, respectively (Figure 3).

### 3.2. CALIPER Functional Analysis

#### 3.2.1. Univariate Analysis

In the derivation cohort, FVC% was not significantly correlated with VRS (*p* = 0.060); however, DLCO% was negatively correlated with VRS (*p* < 0.001). In the validation cohort, both FVC% (*p* = 0.002) and DLCO% (*p* < 0.001) were negatively correlated with VRS.

#### 3.2.2. Multivariable Analysis

In the derivation cohort, multivariable linear correlation showed that total honeycombing (*p* = 0.006) and reticulation (*p* = 0.003), were negatively, and normal lung (*p* < 0.001) volumes were positively correlated with FVC% (Table 4). VRS volume approached statistical significance (*p* = 0.088). In the validation cohort, total reticulation (*p* = 0.017), ground-glass opacity (*p* = 0.039), and normal lung (*p* = 0.009) volumes were correlated with FVC%. Thus, the volumes of total reticulation and normal lung were significantly correlated with FVC% in both cohorts.

In the derivation cohort, multivariable linear correlation showed that total VRS volume was negatively associated with DLCO% (*p* = 0.003), while normal lung volume was positively associated with DLCO (*p* = 0.003) (Table 4). In the validation cohort, total normal lung volume was positively associated with DLCO% (*p* < 0.001), while total VRS (*p* = 0.098) and ground-glass opacity (*p* = 0.079) negatively approached statistical significance. Thus, normal lung correlated with the DLCO in both cohorts.

## 4. Discussion

The purpose of this multicenter study was to assess whether quantitative imaging data from CALIPER were different across CTD-ILD and IPF in the setting of UIP. We demonstrated that only VRS volume was independently and consistently associated with an IPF diagnosis on multivariable analysis. In turn, VRS appeared to be associated with DLCO% but not with FVC%, while total volume of normal lung was associated with both FVC% and DLCO% on multivariable analysis.

Under-recognized morphologic nuances may provide valuable insight into ILD diagnosis [6,7,8]. Quantitative imaging obviates issues of inter-reader and even intra-reader variation in the interpretation of HRCT [9,10,11,23]. The VRS is an intriguing metric that has been increasingly associated with survival and function in ILD patients [12,13,14,15,16,17,18,19]. Our study shows that VRS volume is associated with IPF rather than CTD-ILD, although the mechanism is uncertain. Neovascularization is a well-described phenomenon in pulmonary fibrosis, and this could have contributed to an increased VRS [24,25,26]. VRS may also be a marker of fine perivascular pulmonary fibrosis given VRS’s connection with the extent of ILD [16]. Greater VRS may represent a traction-like phenomenon (“traction vasculectasis”) similar to traction bronchiectasis. Alternatively, VRS may be a marker of pulmonary hypertension, as pulmonary arterial size is a marker of pulmonary hypertension, which is supported by the association between VRS and right ventricular systolic pressure [16,27]. Additionally, this could explain the poor prognosis conferred by higher VRS as pulmonary hypertension is a marker of poor prognosis in ILD [28,29].

VRS appears to be superior to qualitative CT scoring in predicting patient outcomes and function across multiple ILDs [12,13,14,15,16,17,18,19]. VRS may also lend insight into the pathogenesis of IPF as well as factors that contribute to its dismal prognosis. Our data showed that VRS is not associated with FVC%, which suggests that the underlying mechanism of VRS is likely not directly or solely related to the extent of pulmonary fibrosis. On the other hand, VRS is inversely correlated with the DLCO%. It is interesting to note that the only CT-derived measurement that correlated with the FVC% and DLCO% in both cohorts was the quantification of normal lung. The explanation for this might lie in the concept that aside from normal lung, all other CT measurements were surrogates for a pathologic process, which all worked in concert to deleteriously affect lung function.

The typical UIP pattern on CT does not require biopsy for diagnosis given its high positive predictive value for UIP on pathology [30]. However, although UIP is often equated with IPF, UIP may also be secondary to other causes, including CTD-ILD, medications, or inhalational exposures [6,30,31,32,33]. Despite a thorough work-up, some patients with CTD-ILD are misdiagnosed as IPF due to atypical clinical presentations and/or delays in serological conversion. We posit that VRS could be integrated into the diagnostic evaluation of those patients with a typical UIP CT pattern to further inform or risk stratify patients as likely IPF versus CTD-ILD.

Future investigations include validation of our findings in the community setting, where most cases of ILD are managed. In addition, the mechanism underlying VRS’s link to diagnosis, lung function, and outcomes should be explored. Specifically, future research ought to address the question of whether VRS is a marker of vascular disease or due to the effects of fibrosis, akin to traction bronchiectasis (so-called “traction vasculectasis”). In addition, a practical model integrating VRS in the normal work-flow of ILD diagnosis should be more thoroughly explored to assess the incremental improvement in diagnostic accuracy afforded. Future work utilizing artificial intelligence, which does not rely on preconceived models or understanding of underlying mechanisms, is a very promising strategy in this regard.

This study was limited by its retrospective design and the small number of subjects, although the total number of subjects is similar to other studies analyzing quantitative imaging in ILD [14,34,35]. Any bias from the relatively small number of subjects was also mitigated by the dual-center nature and geographic spread of our study population, which suggests that our findings are reliable. However, since the study was performed at two tertiary medical centers specializing in ILD, these results may not be generalizable to the community setting.

VRS appears to have promise in helping predict ILD diagnosis in an additive fashion to the current HRCT interpretative paradigm. Further research is necessary to validate VRS’s signal in ILD across different types of practice settings and diagnoses. Our description of VRS being increased in IPF versus CTD-ILD may provide clues to the differing mechanisms of fibrosis in these two distinct subsets of ILD. Furthermore, the signal in this seemingly insignificant imaging finding suggests that other unrecognized imaging biomarkers might exist that can be leveraged to improve our understanding and care of ILD patients.

## Figures and Tables

**Table 1:** Baseline characteristics stratified by diagnosis at two tertiary medical centers (labeled derivation and validation).

Variable		CTD-ILD (*n* = 40) Derivation	IPF (*n* = 85) Derivation	*p*-Value Derivation	CTD-ILD (*n* = 62) Validation	IPF (*n* = 109) Validation	*p*-Value Validation
Mean years of age		56.9 (15.7)	70.4 (8.0)	**<0.001**	61.9 (13.0)	69.5 (8.3)	**<0.001**
Sex	Female	80.0%	20.0%	**<0.001**	70.0%	10.0%	**<0.001**
	Male	20.0%	80.0%		30.0%	90.0%	
Race	White	65.0%	84.7%	**<0.001**	56.5%	85.3%	**<0.001**
	Black	27.5%	2.4%		25.8%	4.6%	
	Asian	7.5%	5.9%		8.1%	7.3%	
	Other	0.0%	7.0%		6.5%	2.8%	
Median pack-years smoking		21 (20.5)	20 (24)	**<0.001**	0 (22)	20 (28)	**<0.001**
Mean % predicted FVC		61.5 (15.9)	62.1 (16.2)	0.847	63.5 (15.3)	64.1 (18.0)	0.814
Mean % predicted DLCO		40.7 (15.6)	39.7 (14.2)	0.742	45.0 (20.3)	43.9 (17.8)	0.709

Abbreviations: UIP: Usual interstitial pneumonia; TLC = total lung capacity; FVC = forced vital capacity; DLCO = diffusion capacity of the lung for carbon monoxide. Significant *p*-values in bold.

**Table 2:** Univariate CALIPER variables stratified by diagnosis at two tertiary medical centers (labeled derivation and validation).

Variable	CTD-ILD Derivation (*n* = 40)	IPF Derivation (*n* = 85)	*p*-Value Derivation	CTD-ILD Validation (*n* = 62)	IPF Validation (*n* = 109)	*p*-Value Validation
Total honeycombing, median (±IQR)	0.5 (1.3)	2.4 (8.1)	**<0.001**	3.6 (14.3)	7.4 (17.8)	0.087
Total reticulation, median (±IQR)	163.7 (185.8)	193.6 (153.5)	**0.032**	116.9 (106.5)	169.3 (106.5)	**0.003**
Total ground-glass opacity, median (±IQR)	769.5 (649.7)	790.8 (724.9)	0.501	381.0 (550.7)	614.2 (660.9)	**<0.001**
Total VRS, median (±IQR)	114.6 (59.8)	174.7 (84.9)	**<0.001**	142.7 (86.5)	211.3 (78.4)	**<0.001**
Total normal, median (±IQR)	1527.9 (975.5)	1487.8 (923.2)	0.882	1411.8 (814.2)	1764.6 (940.6)	**0.005**

IQR: interquartile range, VRS: vessel-related structures; SD: standard deviation. Significant *p*-values in bold.

**Table 3:** Multivariable logistic regression analysis of CALIPER variables relative to diagnosis across two tertiary medical centers (labeled derivation and validation) for a diagnosis of IPF.

Variable	OR Derivation	95% CI Derivation	*p*-Value Derivation	OR Validation	95% CI Validation	*p*-Value Validation
Total honeycombing	0.995	0.972–1.019	0.687	1.000	0.996–1.004	0.954
Total reticulation	1.002	0.998–1.005	0.327	1.003	0.999–1.006	0.185
Total ground-glass opacity	0.999	0.997–1.000	**0.018**	1.000	0.999–1.002	0.379
Total VRS	1.026	1.014–1.037	**<0.001**	1.008	1.000–1.017	**0.047**
Total normal	1.000	0.999–1.000	0.365	1.001	1.000–1.001	**0.009**
Constant	1.000	0.999–1.000	0.365	0.048	0.011–0.208	**<0.001**

OR: odds ratio; CI, confidence interval; VRS: vessel-related structures. Significant *p*-values in bold.

**Table 4:** Multivariable linear correlation analysis of CALIPER variables relative to percent predicted forced vital capacity (FVC) and diffusing capacity of the lungs for carbon monoxide (DLCO) across two tertiary medical centers (labeled derivation and validation).

Variable	Correlation Coefficient Derivation	SD Derivation	*p*-Value Derivation	Correlation Coefficient Validation	SD Validation	*p*-Value Validation
FVC:						
Total honeycombing	0.132	0.047	**0.006**	0.003	0.016	0.873
Total reticulation	−0.027	0.009	**0.003**	−0.028	0.012	**0.017**
Total ground-glass opacity	0.001	0.003	0.736	−0.008	0.004	**0.039**
Total VRS	−0.043	0.025	0.088	−0.005	0.028	0.859
Total normal lung	0.008	0.002	**<0.001**	0.006	0.002	**0.009**
constant	60.039	5.237	**0.000**	66.125	4.644	**<0.001**
DLCO:						
Total honeycombing	0.020	0.045	0.659	−0.014	0.017	0.395
Total reticulation	−0.007	0.012	0.539	−0.017	0.012	0.176
Total ground-glass opacity	−0.001	0.003	0.786	−0.007	0.004	0.079
Total VRS	−0.079	0.026	**0.003**	−0.048	0.029	0.098
Total normal lung	0.006	0.002	**0.003**	0.009	0.002	**<0.001**
constant	45.435	5.125	**<0.001**	48.161	4.845	**<0.001**

Significant *p*-values in bold.

## Abbreviations

CALIPER	Computer-Aided Lung Informatics for Pathology Evaluation and Rating
CTD-ILD	connective tissue disease-related interstitial lung disease
DLCO	diffusion capacity of the lung for carbon monoxide
DLCO%	percent predicted diffusing capacity of the lungs for carbon monoxide
FVC	forced vital capacity
FVC%	percent predicted forced vital capacity
IPF	idiopathic pulmonary fibrosis
TLC	total lung capacity
UIP	usual interstitial pneumonitis
VRS	vessel-related structure
